# Bacteriophage T4 Vaccine Platform for Next-Generation Influenza Vaccine Development

**DOI:** 10.3389/fimmu.2021.745625

**Published:** 2021-10-12

**Authors:** Mengling Li, Pengju Guo, Cen Chen, Helong Feng, Wanpo Zhang, Changqin Gu, Guoyuan Wen, Venigalla B. Rao, Pan Tao

**Affiliations:** ^1^ Key Laboratory of Development of Veterinary Diagnostic Products, Ministry of Agriculture, College of Veterinary Medicine, Huazhong Agricultural University, Wuhan, China; ^2^ The Cooperative Innovation Center for Sustainable Pig Production, Huazhong Agricultural University, Wuhan, China; ^3^ Division of Pathology, College of Veterinary Medicine, Huazhong Agricultural University, Wuhan, China; ^4^ Hongshan Lab, Wuhan, China; ^5^ Institute of Animal Husbandry and Veterinary Sciences, Hubei Academy of Agricultural Sciences, Wuhan, China; ^6^ Bacteriophage Medical Research Center, Department of Biology, The Catholic University of America, Washington, DC, United States

**Keywords:** flu vaccine, virus-like particle, bacteriophage T4 platform, extracellular domain of matrix protein 2, phage display

## Abstract

Developing influenza vaccines that protect against a broad range of viruses is a global health priority. Several conserved viral proteins or domains have been identified as promising targets for such vaccine development. However, none of the targets is sufficiently immunogenic to elicit complete protection, and vaccine platforms that can enhance immunogenicity and deliver multiple antigens are desperately needed. Here, we report proof-of-concept studies for the development of next-generation influenza vaccines using the bacteriophage T4 virus-like particle (VLP) platform. Using the extracellular domain of influenza matrix protein 2 (M2e) as a readout, we demonstrate that up to ~1,281 M2e molecules can be assembled on a 120 x 86 nanometer phage capsid to generate M2e-T4 VLPs. These M2e-decorated nanoparticles, without any adjuvant, are highly immunogenic, stimulate robust humoral as well as cellular immune responses, and conferred complete protection against lethal influenza virus challenge. Potentially, additional conserved antigens could be incorporated into the M2e-T4 VLPs and mass-produced in *E. coli* in a short amount of time to deal with an emerging influenza pandemic.

## Introduction

Influenza A (Flu) virus is a highly contagious infectious agent that can cause severe respiratory disease (1, 2). Although vaccines are available, they are strain-specific and mainly target the variable head domain of viral major envelope glycoprotein, hemagglutinin (HA) (3, 4). The stalk domain of HA exhibits a degree of conservation among influenza virus strains but cannot efficiently induce antibody responses in its native state due to the immunodominance of epitopes present in the head domain (5–8). The rapid evolution of influenza viruses through antigenic drift and shift in their surface glycoproteins, HA in particular, greatly limit the effectiveness of the current vaccines (9–12). Therefore, vaccines have to be reformulated annually using reference viruses recommended by World Health Organization, based on the information provided by the Global Influenza Surveillance Network (13).

Vaccines that provide broader protection against diverse influenza virus strains are highly desired, and remain as one of the major challenges in Flu vaccine design. Many efforts have been focused on developing such vaccines, often referred to as “universal” Flu vaccines, using the conserved viral proteins or domains (12, 14). The internal virion proteins, nucleoprotein (NP) and matrix protein 1 (M1), were mainly used as immunogens to induce cellular immune responses, particularly CD8+ T cells with cross-protection against heterologous influenza viruses (15–17). Engineered headless HA stalk, in which the immunodominant head domain was removed, was mainly used to induce antibodies that recognize or neutralize diverse influenza virus strains (18, 19). Another widely used target for universal Flu vaccines is the extracellular domain of matrix protein 2 (M2e) that is highly conserved among divergent influenza virus strains (14, 20, 21). However, none of these vaccine targets are highly immunogenic and many strategies were employed to enhance their immunogenicity (22–25).

By taking advantage of *in vitro* assembly of antigen proteins on bacteriophage T4 capsid, we recently developed a virus-like nanoparticle (VLP) platform that can elicit robust immune responses against a variety of displayed antigens, without any adjuvants (26, 27). In this study, we aimed to develop an M2e-based influenza vaccine using this T4 VLP platform. The immunogenicity of M2e, a NH2-terminal 23-residue peptide of viral matrix protein 2 (M2), is otherwise quite low during natural infection due to its small size and low abundance on the virion surface (28). However, when displayed on a VLP, the M2e induced significant immune responses and provided variable protection against influenza virus infection (29, 30). Although many different platforms such as hepatitis B virus core particle (31), human papillomavirus particle (32), and tobacco mosaic virus (33) were used as VLP carriers for M2e, phage-based VLP platforms are more attractive for their cost-effectiveness and large-scale manufacturing potential that is critical during an influenza pandemic.

Several phage platforms have been tested for M2e Flu vaccine design. The M2e antigens displayed on T7 phage capsids, though immunogenic, failed to provide complete protection against lethal influenza virus challenge (34). This was probably due to the low copy number of M2e molecules on the phage capsid. Indeed, it was found that vaccines with higher M2e epitope densities resulted in higher protection efficacy (35, 36), and most of the licensed viral vaccines contain high density of antigens on the virion surface (37). Although phage fd can display up to 2,700 copies of peptide per capsid through its major coat protein pVIII, the display is sensitive to the size of the peptide (38). Therefore, only part of M2e (residues 2-16) could be displayed on phage fd, which still provided protection from death but the challenged mice showed severe body weight loss indicating its limited value as a vaccine (39).

**Figure 1:**
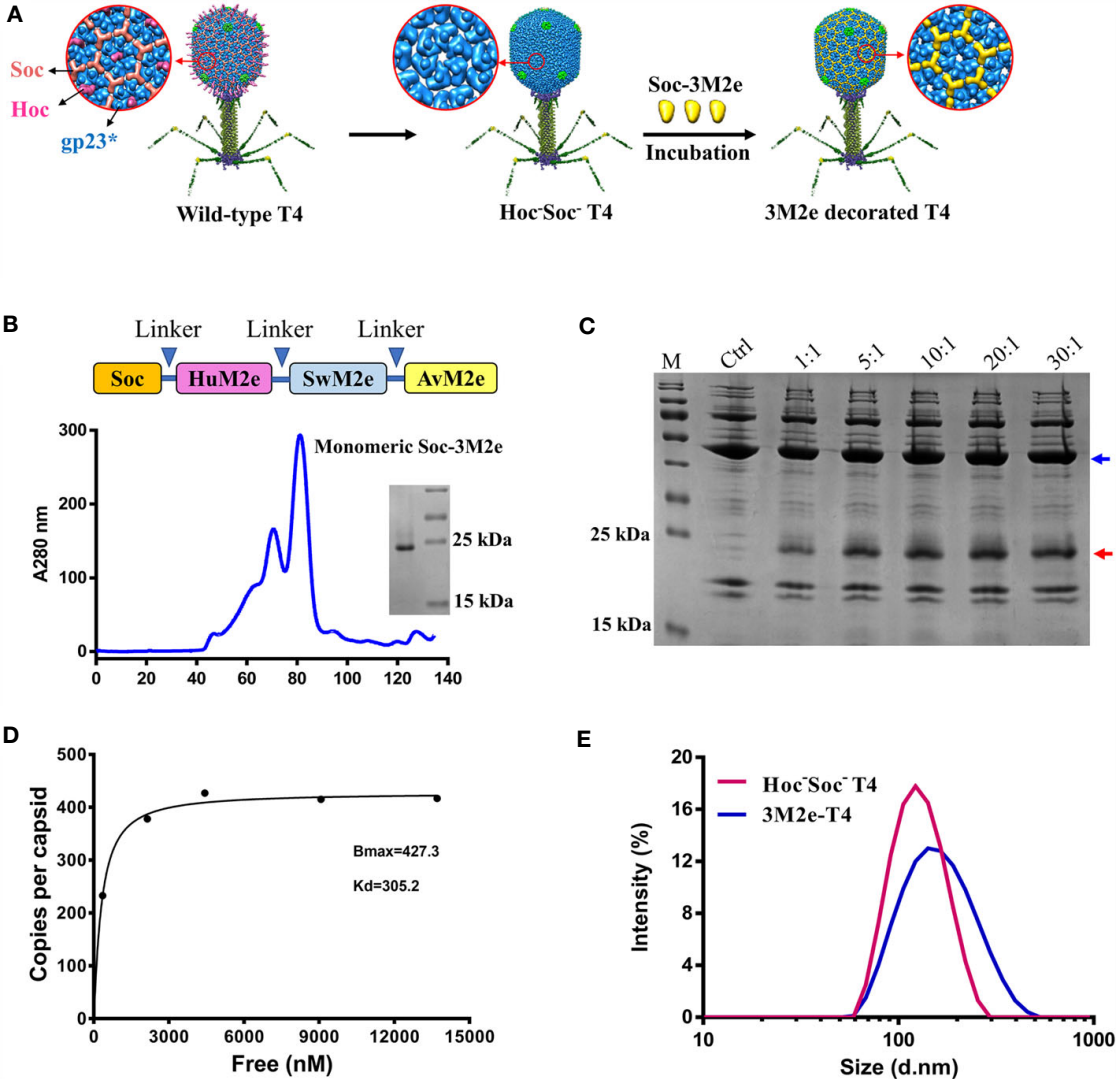
Construction of 3M2e-T4 VLPs. **(A)** schematic diagram of 3M2e-T4 VLPs preparation. Structural models of wild-type T4 and Hoc^-^Soc^-^ T4 phages were shown. 3M2e decorated T4 nanoparticles were prepared by incubation of Soc-3M2e proteins with Hoc^-^Soc^-^ T4 phages as described in Materials and Methods. **(B)** Purification of Soc-3M2e. Soc-3M2e fusion was constructed by fusing 3M2e, which contains three tandem copies of M2e from human, swine, and avian influenza viruses, to the COOH-terminus of Soc. Arrows indicated the flexible linkers (GGSSGGSS) between each component. Soc-3M2e protein was purified by HisTrap affinity chromatography followed by size exclusion chromatography. Only the monomeric peak was collected, and the purity of Soc-3M2e protein was analyzed by SDS-PAGE. **(C)** Assembly of 3M2e-T4 VLPs *in vitro*. About 5×10^10^ Hoc^-^Soc^-^ T4 phages were incubated with at the indicated ratios of Soc-3M2e protein molecules to capsid binding sites (see Materials and Methods for the details). 3M2e-T4 VLPs were analyzed by SDS-PAGE. The same amount of Hoc^-^Soc^-^ T4 phages was used as a control. Blue and red arrows indicated gp23* and Soc-3M2e, respectively. **(D)** Saturation binding curve of Soc-3M2e. The bound and unbound (not shown) Soc-3M2e proteins were calculated using BSA a standard. The copy numbers of Soc-3M2e per capsid were determined using gp23* as internal control. The data were plotted as one-site saturation ligand binding curve. **(E)** The diameter distributions of Hoc^-^Soc^-^ T4 (red line) and 3M2e-T4 nanoparticles (blue line).

We have previously reported that phage T4 can be used for efficient display of full-length proteins as large as 120 kDa at high density because of its unique capsid architecture (40). The 120 x 86 nm phage capsid is comprised of four major capsid proteins: two essential proteins, the capsid shell protein gp23* (930 copies) and the vertex protein gp24* (55 copies), and two non-essential proteins, the small outer capsid protein (Soc, 870 copies) and the highly antigenic outer capsid protein (Hoc, 155 copies) (**Figure 1A**) (26, 41). Deletion of Hoc and Soc (Hoc^-^Soc^-^ T4) has no effect on the propagation of T4 under laboratory conditions, and recombinant antigens fused to Hoc or Soc specifically bind to Hoc^-^Soc^-^ T4 capsids *in vitro* with high affinity (26, 27, 42, 43).

Here, we show that three variants of M2e peptide from human, swine, and avian influenza viruses tandemly fused to the COOH-terminus of Soc can be efficiently displayed on Hoc^-^Soc^-^ T4 capsids at high density by *in vitro* assembly (**Figure 1**). The resultant M2e-decorated T4 nanoparticles are found to be highly immunogenic and induced complete protection against lethal influenza virus challenge, without any adjuvant. Importantly, the vaccinated mice showed no or minor symptoms after lethal influenza virus challenge, based on clinical observations including body weight and pathological analyses. These studies provide proof-of-concept for the development of next-generation influenza vaccines using the phage T4 VLP platform.

## Results

### Construction of M2e-Decorated Bacteriophage T4 Nanoparticles

To stimulate increased breadth of immunogenicity and protection against Flu viruses, a 3M2e gene containing three types of M2e sequences from human, swine, and avian influenza viruses were synthesized (**Supplementary Table**) and fused to the COOH-terminus of Soc to generate Soc-3M2e. A flexible GGSSGGSS linker was introduced between each M2e segment as depicted in **Figure 1B**, to minimize any interference in the folding of the M2e domains. Hexa-histidine tags were also added to both termini of the Soc-3M2e protein. The fusion protein was expressed in *E. coli* and purified by Ni^2+^ affinity chromatography followed by size-exclusion chromatography. The major peak corresponding to a molecular weight of ~21.8 kDa (monomeric Soc-3M2e) was collected (**Figure 1B**, blue profile). The purity of the recombinant Soc-3M2e proteins was confirmed by sodium dodecyl sulfate–polyacrylamide gel electrophoresis (SDS-PAGE), which showed a single major band with a molecular mass of ~22 kDa, equivalent to the Soc-3M2e fusion protein (**Figure 1B**).

The 3M2e-decorated T4 nanoparticles (3M2e-T4 nanoparticles) were prepared by incubation of the purified Soc-3M2e protein with the CsCl-purified Hoc^-^Soc^-^ T4 phages as previously described (44) (**Figure 1C**). To optimize the copy number of 3M2e, ~5×10^10^ T4 phages were incubated with different quantities of the Soc-3M2e protein (**Figure 1C**). The presence of bound Soc-3M2e was determined by SDS-PAGE analysis of 3M2e-T4 nanoparticles isolated by high-speed centrifugation. The data show that the Soc-3M2e protein bound efficiently to the Hoc^-^Soc^-^ T4 phages, even at a 1:1 ratio of Soc-3M2e molecules to Soc binding sites, and reached saturation at ratio of 10:1 (**Figure 1C**). The copy number of bound Soc-3M2e per capsid (B_max_) calculated from the binding curve was 427, and the apparent binding constant (K_d_) was 305 nM (**Figure 1D**). Since each Soc-3M2e protein contains three tandem copies of M2e peptide, there are ~1,281 M2e molecules assembled on each T4 nanoparticle, which is remarkably higher than any VLPs reported so far. The diameter distribution and zeta-potential of 3M2e-T4 nanoparticles was determined using Zetasizer Nano ZS. The average diameter of 3M2e-T4 nanoparticles is 150.9 nm, which is larger than the diameter of Hoc^-^Soc^-^ T4 phages (mean = 120.8 nm) (**Figure 1E**), indicating the binding of Soc-3M2e. We didn’t observe significant difference in zeta-potentials between Hoc^-^Soc^-^ T4 (-26.7 ± 0.4 mV) and M2e-T4 nanoparticles (-24.3 ± 0.12 mV) (**Figure S1**).

### 3M2e-T4 Phage Nanoparticles Induced Robust M2e-Specific Antibodies

**Figure 2:**
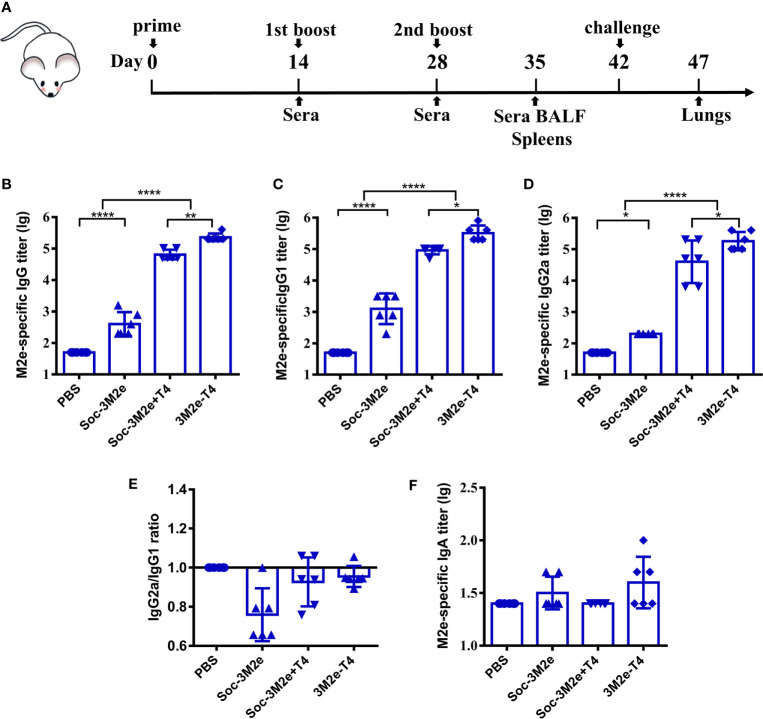
M2e-specific humoral immune responses. **(A)** Scheme of mouse immunization. Sera were obtained before each immunization. The titers of M2e-specific IgG **(B)**, IgG1**(C)**, IgG2a **(D)**, and IgA **(F)** were determined by ELISA using peptides pool of M2e from human, swine, and avian influenza viruses. **(E)** Ratio of M2e-specific IgG2a to IgG1 were calculated. Date were shown as means ± S.D. *, **, and **** indicated p < 0.05, p < 0.01, and p < 0.0001 respectively (ANOVA).

To determine the immunogenicity of 3M2e-T4 nanoparticles, mice were intramuscularly immunized with 3M2e-T4 nanoparticles displaying a total of 15μg 3M2e antigen on day 0, 14, and 28 (**Figure 2A**). Mice immunized with PBS, 15μg of Soc-3M2e soluble proteins, or a simple mixture of 15μg Soc-3M2e antigen and the same number of T4 phage nanoparticles (Soc-3M2e+T4) were used as controls. To minimize binding prior to immunization, the Soc-3M2e antigen was mixed with phage T4 at the time of immunization (most of the Soc-3M2e did not bind to capsid, see Materials and Methods for the details). Sera were collected according to the scheme shown in **Figure 2A**, and the titers of M2e-specific antibodies were determined by enzyme-linked immunosorbent assay (ELISA). All mice immunized with PBS were negative for M2e-specific IgG even at a low sera dilution of 50. The Soc-3M2e soluble proteins induced very low levels of M2e-specific IgG antibodies. However, 3M2e-T4 nanoparticles), without any adjuvant, induced very high levels of 3M2e-specific IgG antibodies, with end point titers of ~4×10^5^ (**Figure 2B**). Even a simple mixture of Soc-3M2e and T4 phage (Soc-3M2e+T4 mixture) generated high titers (~1×10^5^) of M2e-specific antibodies when compared to the very low titers generated by the soluble Soc-3M2e antigen (**Figure 2B**, p<0.0001, ANOVA). These data point to the remarkable immune stimulatory effect of the T4 phage nanoparticles.

Since M2e-induced immune protection mainly depends on antibody-dependent cellular cytotoxicity (ADCC) and antibody-dependent cellular phagocytosis (ADCP), the efficiencies of which are different between IgG subtypes (45–47), we determined the titers of M2e-specific IgG1 (T_H_2-biased) and IgG2a (T_H_1-biased). The Soc-3M2e soluble antigen mainly induced IgG1 antibodies, whereas mice immunized with 3M2e-T4 nanoparticles or Soc-3M2e+T4 mixture produced similar levels of both IgG1 and IgG2a antibodies (**Figures 2C**–**E**). Elicitation of balanced T_H_1 and T_H_2 biased immune responses is a significant feature of the T4 vaccine delivery platform and generally important for protection against infectious disease. We have also determined the 3M2e-specific IgA antibodies in sera. Although 3M2e-T4 nanoparticles induced IgA antibodies in some of the mice, the data were not statistically significant (p>0.05, ANOVA. **Figure 2F**).

### 3M2e-T4 Nanoparticle-Induced Anti-M2e Antibodies Bind to Influenza Virions and Influenza Virus-Infected Cells

**Figure 3:**
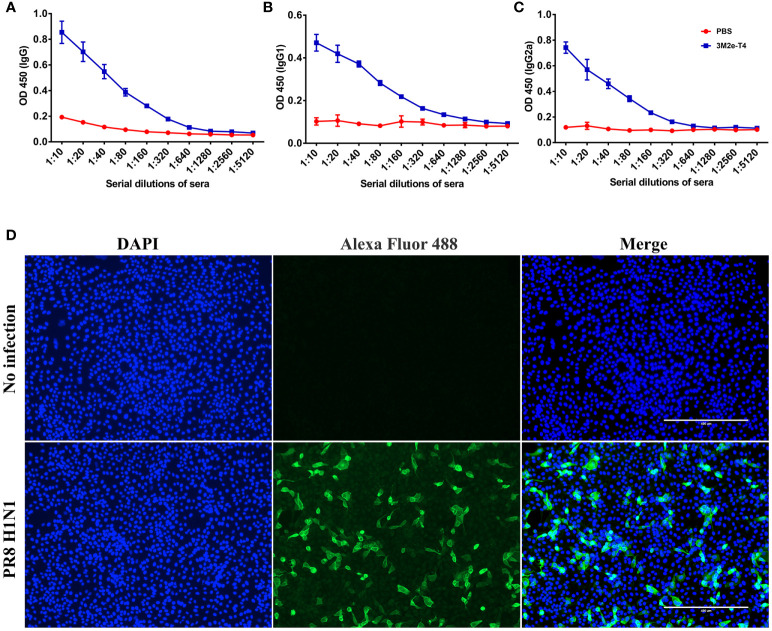
Anti-M2e antibodies specifically bind to influenza virions and influenza virus-infected cells. The binding of M2e-specific IgG **(A)**, IgG1**(B)**, and IgG2a **(C)** to influenza virions were determined by ELISA using β-propiolactone-inactivated A/Puerto Rico/8/34 virus as the coating antigen. **(D)** The binding of M2e-specific IgG to influenza virus infected cells. MDCK cells were infected with A/Puerto Rico/8/34 virus at a MOI of 1, and the binding was determined by indirect immunofluorescence assay as described in Materials and Methods. Data were shown as means ± S.D.

To determine whether 3M2e-T4 nanoparticles induced antibodies recognize M2e on influenza virions, A/Puerto Rico/8/34 (H1N1) virus was inactivated with β-propiolactone and used as the coating antigen in ELISA assays. The data revealed that these antibodies, regardless of the IgG subtype, specifically bound to the influenza virions (**Figures 3A**–**C**). Consistent with the end point titers (**Figure 2**), balanced levels of virion-binding titers were observed for both the IgG1 and IgG2a subtypes. As expected, sera from PBS immunized mice showed negative results even at a low sera dilution of 10 (**Figures 3A**–**C**). Since M2e-induced immune protection mainly depends on ADCC and ADCP, we then examined whether M2e presented in plasma membranes of influenza virus-infected cells can be recognized by 3M2e-T4 induced antibodies. Madin-Darby canine kidney (MDCK) cells infected with A/Puerto Rico/8/34 (H1N1) influenza virus at a multiplicity of infection (MOI) of 1 were tested for the binding of M2e-specific antibodies by indirect immunofluorescence assay (**Figure 3D**). The data indicated that the sera from 3M2e-T4 VLPs immunized mice showed significant binding to influenza virus-infected cells, but not to mock-treated cells (**Figure 3D**). Together, these data demonstrated that the 3M2e-T4 nanoparticle can efficiently induce M2e-specific antibodies that recognize M2e presented both on influenza virions and on virus-infected cells, indicating their potential protection against influenza virus infection.

### 3M2e-T4 Nanoparticles Elicit Strong Cellular Immune Responses

**Figure 4:**
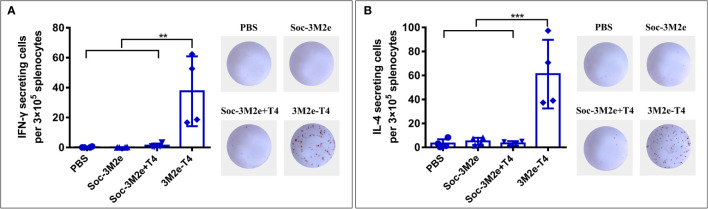
M2e-specific cellular immune responses. Mice (n = 4) were immunized according to the scheme shown in **Figure 2A**, and splenocytes were isolated on day 35. The IFN-γ **(A)** and IL-4 **(B)** secreting lymphocytes were assayed by ELISPOT as described in Materials and Methods. The right images of each panel show the representative results of ELISPOT wells from each group. Data were represented as mean ± S.D. of four mice in each group. **p < 0.01; ***p < 0.001 (ANOVA).

To investigate whether the 3M2e-T4 nanoparticles elicited M2e-specific cellular immune responses, mice were sacrificed 7 days after the second boost, and spleens were collected to isolate peripheral blood mononuclear cells (PBMCs). The number of IFN-γ and IL-4 secreting cells were analyzed by ELISPOT using 10μg/ml M2e peptide as a stimulus. Neither the mice immunized with Soc-3M2e soluble antigen nor those immunized with the Soc-3M2e+T4 mixture generated IFN-γ and IL-4 secreting cells, while the mice immunized with 3M2e-T4 nanoparticles generated significant numbers of M2e-specific IFN-γ (**Figure 4A**) and IL-4 secreting cells (**Figure 4B**). These data demonstrate that assembly of 3M2e on T4 nanoparticles is essential to stimulate the cellular arm of the host immune system. This seems to be not that critical for the humoral arm because, as shown above, substantial induction of antibodies was evident with the Soc-3M2e+T4 mixture (**Figures 2B**–**D**).

### 3M2e-T4 Nanoparticles Elicited M2e-Specific Mucosal Antibodies

**Figure 5:**
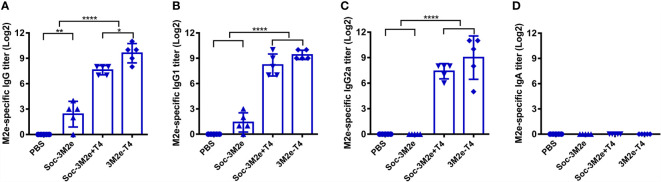
M2e-specific antibodies in BALF. Bronchoalveolar lavage fluids were collected 7 days after last immunization (n = 5). M2e-specific total IgG **(A)**, IgG1**(B)**, IgG2a **(C)**, and IgA **(D)** were determined by ELISA using a mixture of human, swine, avian influenza virus M2e peptides as the capture antigen (2 µg/ml). Date were presented as means ± S.D. *p < 0.05; **p < 0.01; ****p < 0.0001 (ANOVA).

The mucosal surfaces of the respiratory tract are major ports of entry for influenza viruses, and previous studies indicated that both mucosal IgG and serum IgG are conducive for efficient protection (48). To determine if the 3M2e-T4 nanoparticles stimulated mucosal antibodies, bronchoalveolar lavage fluid (BALF) was collected 7 days post last immunization, and the presence of IgG and IgA antibodies were detected using ELISA. As shown in **Figure 5A**, soluble Soc-3M2e was able to induce low levels of M2e-specific IgG antibodies, whereas the 3M2e-T4 nanoparticles induced the highest levels. As in the case of serum IgG, the Soc-3M2e+T4 mixture could also induce mucosal IgG, much higher than the soluble antigen. As expected, the PBS control mice had no detectable levels of anti-M2e IgG antibodies (**Figure 5A**). Subtype analysis of the IgG antibodies showed that Soc-3M2e soluble antigens only elicited M2e-specific mucosal IgG1, whereas mice immunized with 3M2e-T4 nanoparticles or Soc-3M2e+T4 mixture induced balanced levels of both mucosal IgG1 and IgG2a antibodies (**Figures 5B, C**). However, all the groups failed to develop M2e-specific IgA antibodies in BALF (**Figure 5D**).

### 3M2e-T4 Nanoparticles Provided Complete Protection Against Influenza A Virus Challenge

**Figure 6:**
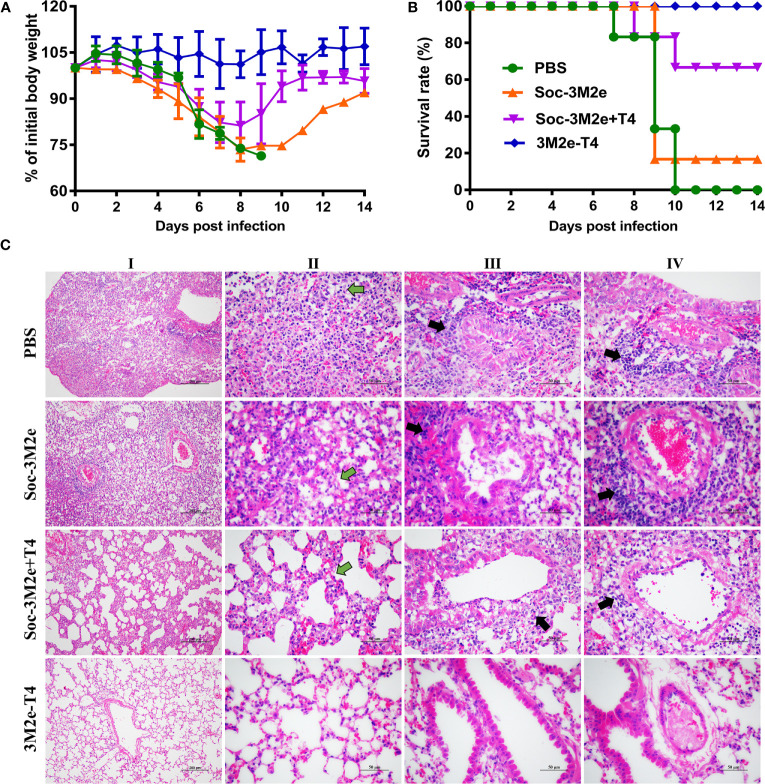
3M2e-T4 VLPs provided complete protection against influenza virus challenge. Mice (n = 6) were challenged with 5×LD_50_ of A/Puerto Rico/8/34 two weeks after last immunization. Weight loss **(A)** and survival rate **(B)** of mice were monitored daily for 14 days. **(C)** Pathological analysis of lungs from mice (n = 3) challenged with virus was carried out in a separate experiment, in which mice were immunized with the same immunization procedure described above. Five days post infection with 5×LD_50_ of A/Puerto Rico/8/34, mice were euthanized, and lung sections were prepared as described in Materials and Methods. The representative results from each group were shown (column I, scale bar, 200 µm; Columns II-IV, scale bar, 50 µm). Main pathological changes are thickening of alveolar septa of mice (column II, green arrows), inflammatory cells infiltrated around the bronchi (column III, black arrows), and pulmonary blood vessels (column IV, black arrows).

To evaluate the protective efficacy of each formulation, immunized mice were challenged with 5LD_50_ of A/Puerto Rico/8/34 (H1N1) virus and monitored daily for body weight and survival for 14 days. As shown in **Figure 6A**, infection of influenza virus resulted in substantial weight loss of PBS control mice, or mice immunized with either the Soc-3M2e soluble antigen, or Soc-3M2e+T4 mixture, three days post infection. All the mice in the PBS control group died 10 days post challenge and five of the six mice immunized with the soluble Soc-3M2e died 9 days post challenge (**Figure 6B**), whereas 67% of the mice vaccinated with Soc-3M2e+T4 mixture recovered and survived (**Figure 6B**). In contrast, all the mice immunized with 3M2e-T4 nanoparticles not only survived the lethal challenges with the H1N1 virus infections (**Figure 6B**), but also, remarkably, showed no significant body weight loss (**Figure 6A**).

The protection efficacy was further evaluated by pathological analysis of the lungs of the immunized mice 5 days post-challenge. **Figure 6C** shows representative results of the lung lesions (column I) and pathological changes of alveoli (column II), bronchi (column III), and pulmonary vessels (column IV). Overall, mice immunized with Soc-3M2e+T4 mixture showed obvious, though less severe, lesions in the lungs when compared to the PBS control mice or mice immunized with soluble Soc-3M2e antigen, whereas no obvious lesions were found in 3M2e-T4 nanoparticle immunized mice (column I). The alveolar walls of PBS or soluble Soc-3M2e immunized mice were severely thickened (column II), and a large number of inflammatory cells infiltrated around the bronchi (column III) and pulmonary blood vessels (column IV). Mice immunized with Soc-3M2e+T4 mixture also exhibited similar but less severe pathological changes. However, in contrast, the mice vaccinated with 3M2e-T4 nanoparticles showed relatively normal alveolar wall thickness and negligible inflammatory infiltration (**Figure 6C**).

## Discussion

The influenza virus M2e antigen is considered to be an attractive target for the development of universal influenza vaccines (49–51). However, VLP carriers and/or adjuvants are absolutely needed for designing the M2e-based vaccines because of the poor immunogenicity of the M2e peptides. By taking advantage of the phage T4 nanoparticle platform, in this study, we developed a novel 3M2e-T4 VLP vaccine that, without any adjuvant, induced robust humoral and cellular immune responses and provided complete protection against influenza virus challenge.

The 3M2e-T4 nanoparticles were prepared by simply incubating the Soc-3M2e fusion protein with Hoc^-^Soc^-^ T4 phages, both of which can be produced in large-scale in *E. coli*. Therefore, our M2e-T4 nanoparticles provide an approach to manufacture influenza vaccines in a short amount of time, which is critical to deal with an emerging influenza pandemic. The 3M2e-T4 nanoparticles can be stored at 4°C for at least 6 days without significant degradation of the bound antigen (**Figure S2**), and it can also be lyophilized for long-term storage. Although phages T7 and fd have previously been used as carriers to present M2e, they conferred limited protection (34, 39). This is probably because these platforms cannot present full-length M2e peptides at high density, which is a key determinant for inducing strong and protective immune responses (52). Our data demonstrated that each T4 phage nanoparticle was decorated with ~427 copies of the 21.8 kDa Soc-3M2e, and each copy containing three tandem repeats of M2e peptide from human, swine, and avian influenza viruses. This means that ~1,281 copies of M2e molecules were presented on a 120 x 86 nm nanometer capsid particle, the highest density reported so far on any VLP. Such high density as well as the repetitive and symmetrical arrangement of M2e epitopes mimicking the surface structure of a viral pathogen, probably led to robust stimulation of the host immune system eliciting strong immune responses without the need for an adjuvant.

Apart from high epitope density, the high immunogenicity of 3M2e-T4 VLPs might also because the T4 phage was able to stimulate innate immune responses and may have natural adjuvant properties (26, 38). Indeed, a mixture of Soc-3M2e antigen and T4 phages (Soc-3M2e+T4) in which most of the antigen was not attached to capsid induced quite high levels of M2e-specific antibodies in sera (**Figures 2B**–**D**) and BALF (**Figure 5**). Importantly, however, the 3M2e-T4 nanoparticles elicited the strongest immune responses. This might be because display of 3M2e on T4 phage represents linkage of antigen to an adjuvant-loaded delivery system, which ensures simultaneous presentation of both to the same immune cell such as the antigen-presenting cells (APCs) that could significantly enhance the immune responses (53). T4 phages also induced vector-specific antibodies after immunization (**Figure S3A**), which could be a concern of T4 VLP platform. However, such antibodies did not interfere the immunogenicity of 3M2e displayed on T4 nanoparticles based on the observation that M2e-specific antibodies were boosted significantly after second and third immunizations (**Figure S3B**). This might because T4 phage is mainly used as a scaffold to efficiently deliver and present antigens to immune system, and it cannot replicate in mice as some other live viral vectors. The efficacy of such live viral vaccines depends on their replication *in vivo* to produce enough antigens, which might be inhibited by vector-specific immune responses. Our data indicated that the T4 vector immunogenicity is not a significant issue in the case of T4 VLP vaccines.

Unlike soluble Soc-3M2e protein that mainly elicited Th2-biased responses, the 3M2e-T4 VLPs induced balanced T_H_1 and T_H_2 immune responses (**Figures 2E**, **4**), which is vital for vaccine efficacy. T_H_1-type cytokines such as IFN-γ tend to induce the proinflammatory responses, which could lead to tissue damage. T_H_2-type cytokines such as IL-4 are mostly involved in mediating anti-inflammatory response, which will counteract the excessive microbicidal effect mediated by T_H_1-based responses (54–56). Our results showed that mice immunized with 3M2e-T4 VLPs, but not soluble Soc-3M2e proteins, induced similar levels of M2e-specific IgG1 and IgG2a (**Figure 2E**). Similar results were also observed for the M2e-specific IFN-γ and IL-4 secreting cells (**Figure 4**) indicating that M2e-T4 VLPs facilitate both T_H_1-type and T_H_2-type immune responses. Additionally, M2e-specific antibodies generally are non-neutralizing and their protection is mainly dependent on antibody-dependent cellular cytotoxicity (ADCC) and antibody-dependent cellular phagocytosis (ADCP) (45–47, 57). Therefore, high levels of M2e-specific IgG2a antibodies, which are more potent than other IgG subclasses in directing ADCC, are desirable for M2e-based vaccines.

Previous studies have suggested that T-cell responses induced by M2e vaccines also contributed to the protection against influenza infections (49, 58). In our current study, we found that the 3M2e-T4 VLPs, but not the soluble Soc-3M2e or a mixture of T4 and Soc-3M2e, elicited high levels of M2e-specific IFN-γ and IL-4 secreting lymphocytes in the spleens of vaccinated mice. These might have contributed to the enhanced protections of 3M2e-T4 VLPs (**Figure 6**), and the expectation is that, since we have used three versions of M2e and that M2e as such is highly conserved among influenza viruses, these responses would afford cross-protection to diverse viruses belonging to different subtypes.

Although M2e-based universal influenza vaccines are promising, it is highly desirable to include other conserved antigens such as HA-stalk, NP, and M1 to cover a broad range of virus types. Other than displaying M2e on capsids, our T4 vaccine platform provides flexibility and capacity for next-generation multivalent vaccine design. For instance, we have demonstrated that the T4 platform can be used to simultaneously display and deliver different kinds of protein antigens or DNAs encoding antigen proteins, target antigens to dendritic cells, and co-deliver antigens and molecular adjuvants (27, 38, 40). Therefore, potent multivalent T4-Flu VLP vaccines can be designed and experiments are currently underway to develop such next-generation broadly effective Flu vaccines.

In conclusion, our studies demonstrated that the T4 phage nanoparticles displaying the Flu viral M2e peptides at high density, without the inclusion of an external adjuvant, stimulate strong humoral and cellular immune responses in mice against the virion-exposed M2e that is otherwise poorly immunogenic. These responses also afforded complete protection against lethal Flu virus challenge. These results, thus, provide a proof-of-concept for the development of potent next-generation influenza vaccines using the T4 VLP platform by incorporating additional conserved influenza antigens and other immunostimulatory molecules.

## Materials and Methods

### Ethics Statement

All animal experiments were approved by the Research Ethics Committee (HZAUMO-2021-0023), Huazhong Agricultural University, Hubei, China and performed in the Laboratory Animal Center of Huazhong Agricultural University strictly in accordance with the Guidelines for the Care and Use of Laboratory Animals, Huazhong Agricultural University.

### Construction of Plasmids

The plasmid pET-RbSoc was constructed by inserting Soc gene of RB69 phage into pET28b expression vector using NheI and XhoI restriction sites. The 3M2e gene (**Table S1**) encoding three tandem copies of M2e from human, swine, and avian influenza viruses separated by two flexible linkers (GGSSGGSS) was synthesized and cloned into pUC19 vector using SalI and XhoI restriction sites. The 3M2e gene fragment was cut from pUC19 plasmid using SalI and XhoI and subcloned into pET-RbSoc plasmid at the XhoI site to generate the expression plasmid pRbSoc-3M2e, in which the 3M2e gene was fused to COOH-terminus of RB69 Soc.

### Purification of Recombinant Soc-3M2e Protein

The pRbSoc-3M2e expression plasmid was transformed into *E. coli* BL21 (DE3) competent cells (Novagen), and a single colony was cultured overnight in LB medium supplemented with 50 µg/ml kanamycin. Ten ml of the overnight culture was inoculated into 1 L of fresh LB medium containing the antibiotic, and the expression of Soc-3M2e was induced with 1mM isopropyl-β-D-thiogalactoside (IPTG) at 30°C for 2 hours when the OD600 of culture reached 0.8. *E. coli* cells were collected by centrifugation at 4,300g for 15 min and resuspended with binding buffer (20 mM Tris-HCl pH 8.0, 100 mM NaCl, 10 mM imidazole, and 5 μg/ml DNase I). The cells were lysed by high-pressure cell disruptor at 4°C, and cell debris was removed by high-speed centrifugation (35,000g, 20 min, 4°C). The supernatant containing the recombinant Soc-3M2e proteins was passed through a 0.22 μm filter and loaded onto HisTrap column (Yeasen, Shanghai, China). After washing with 40 ml washing buffer (20 mM Tris-HCl pH 8.0, 100 mM NaCl, and 20 mM imidazole), the Soc-3M2e protein was eluted with elution buffer (20 mM Tris-HCl pH 8.0, 100 mM NaCl, and 400 mM imidazole). The peak fractions were collected and further purified by size-exclusion chromatography (Hi-load 16/60 Superdex 200 column, GE Healthcare Life Sciences) using gel filtration buffer (20 mM Tris-HCl pH 8.0, 100 mM NaCl). The purity of eluted Soc-3M2e protein was analyzed by SDS-PAGE, and protein concentration was determined with BSA as a standard. The endotoxin level present in purified Soc-3M2e protein was 0.05 EU/ml tested using Limulus Amebocyte Lysate (LAL) Endotoxin Quantitation Kit (Xiamen Bioendo Technology Co., Ltd, Xiamen China).

### T4 Phage Purification

The propagation and purification of Hoc^-^Soc^-^ phage T4 were carried out as previously described (44, 59, 60). Briefly, an overnight culture of *E. coli* P301 was inoculated into LB/M9CA medium and incubated at 37°C until the cell density reaches 1.5-2.0×10^8^ cells/ml. *E. coli* cells were then infected with Hoc^-^Soc^-^ phage T4 at a multiplicity of infection (MOI) of 0.2-0.4, and cultured at 37°C for another 2-3 h. The cultures were harvested by centrifugation at 30,000g for 30 min, and the pellet containing phages was suspended in Pi-Mg buffer (26 mM Na_2_HPO_4_, 22 mM KH_2_PO_4_, 79 mM NaCl, 1 mM MgSO_4_) containing chloroform and DNase I. After 20 min incubation at 37°C, the suspension was centrifuged at 4,300g for 20 min to remove cell debris, and phages in the supernatant were collected by high-speed centrifugation (30,000g, 30 min). The phage pellet was resuspended in 1 ml Pi-Mg buffer and purified by CsCl step density gradient centrifugation. Finally, the phages were dialyzed against dialysis solution I (10 mM Tris pH 8.0, 200 mM NaCl, and 5 mM MgCl_2_) for 5 h followed by dialysis solution II (10 mM Tris pH 8.0, 50 mM NaCl, and 5 mM MgCl_2_) for overnight at 4°C. The endotoxin level present in purified Hoc^-^Soc^-^ phages was 1.53 EU/ml, which is well under the maximum recommended endotoxin levels, 20 EU/ml, in subunit vaccines (61).

### Assembly of 3M2e Antigens on Hoc^-^Soc^-^ T4 Capsids *In Vitro*

*In vitro* assembly of proteins on Hoc^-^Soc^-^ T4 phages was performed as described previously (26, 44). To optimize the binding of Soc-3M2e, about 5 × 10^10^ phage particles were incubated at 4°C for 45 min with Soc-3M2e proteins at different ratios of antigen molecules to Soc binding sites (1:1 to 30:1, **Figure 1C**). The unbound Soc-3M2e proteins were removed by centrifugation at 21,130 g for 30 min, and the pellet of phage particles containing bound proteins were washed twice with PBS. The Soc-3M2e decorated phage particles were finally resuspended in PBS, transferred to a new tube, and analyzed by SDS-PAGE. The gp23* and Soc-3M2e proteins in each lane were quantified by Image-Pro Plus software using BSA as a standard. The copy number of bound Soc-3M2e proteins was determined using gp23* (49 kDa, 930 copies per capsid) as internal control. The saturation binding curve was generated using Prism GraphPad software as previously described (26, 27, 43). The y-axis showed the copy number at the different ratios, while x-axis represented the concentration of unbound Soc-3M2e. The apparent binding affinity (K_d_) and the maximal number of bound molecules (B_max_) were determined by nonlinear regression analysis with the methods of one site specific binding with a Hill slope. The diameter distribution and zeta-potential of the nanoparticles were determined using Zetasizer Nano ZS (Malvern Panalytical, UK).

### Immunizations and Influenza A Virus Challenge

Six- to eight-week-old female BALB/c mice were purchased from Laboratory Animal Center of Huazhong Agricultural University, Hubei, China. The 3M2e-T4 nanoparticles were prepared as described above and injected intramuscularly into mice (15μg 3M2e per dose) at week 0, 2, and 4. Each batch of sample was analyzed by SDS-PAGE for consistency in the copy number of displayed antigens before injection (**Figure S4**). Mice immunized with the mixture of Soc-3M2e proteins (15μg 3M2e per dose) and T4 phages (9×10^11^ particles) were used as controls (Soc-3M2e+ T4). To minimize the binding of Soc-3M2e, the protein was mixed with T4 phages right before each immunization. Other control groups included PBS, Soc-3M2e soluble proteins (15μg 3M2e per dose), and T4 phages (9×10^11^ particles). Two weeks post the third immunization, mice (n=6) were anaesthetized with ether and intranasally infected with 5 LD_50_ of influenza A/Puerto Rico/8/1934 virus. All mice were monitored daily for morbidity and mortality for 14 days. Animals with 30% or greater body weight loss were euthanized immediately and considered as death.

### Quantification of Antibodies in Sera and BALF

The levels of antigen-specific antibodies in sera and bronchoalveolar lavage fluid (BALF) were quantified by enzyme-linked immunosorbent assay (ELISA). Blood samples (n=6) were collected on days 0, 14, 28, and 35. BALF samples (n=5) were collected 7 days after the third immunization. Briefly, the lungs from sacrificed mice were flushed three times with 1ml PBS, which was then centrifugated at 3,500 g for 10 min. The supernatant was harvested for analysis of M2e-specific antibody titers. The ELISA plates were coated overnight at 4°C with 200 ng/well of M2e peptide pool consisting of equal amounts of peptides from human, swine, and avian influenza viruses or β-propiolactone-inactivated influenza A virus (1×10^6^ PFU/well) or Hoc^-^Soc^-^ T4 phages (1×10^9^ PFU/well). The plates were blocked with 3% BSA in PBS-T (PBS containing 0.05% Tween-20) for 1 hour at 37°C. Serially diluted sera and BALF (1% BSA in PBS-T) were then added to each well of plates, which were incubated at 37°C for 1 hour. After washing 5 times with PBS-T, the plates were incubated with secondary antibodies (horseradish peroxidase-conjugated goat anti-mouse IgG, IgG1, IgG2a, and IgA) at 37°C for 1 hour. Following five washes, 100 μl of TMB (3,3′,5,5′-tetramethylbenzidine) substrate was added to each well, and the reaction was stopped with 2 M H_2_SO_4_. The absorbance atOD450 was determined by a microplate reader.

### Indirect Immunofluorescence Assay

About 2×10^5^ MDCK cells in 500 μl of growth medium (DMEM supplemented with 10% fetal bovine serum) were seeded into an each well of 24-well plate and allowed to adhere overnight. The growth medium was removed, and cells were washed twice with PBS and mock infected or infected with A/Puerto Rico/8/1934 virus in serum-free DMEM at a MOI of 1 for 1 h. The cells were then washed three times with PBS and cultured in serum-free DMEM containing 1 μg/ml TPCK-trypsin for 20h. After washing with PBS, cells were fixed with 10% formalin for 10 min, permeabilized with 0.1% Triton X-100 for 20 min, and blocked with 5% BSA in PBS-T (PBS containing 0.05% Tween-20) for 1 hour at 37 °C. The cells were then incubated with the sera from immunized mice at a dilution of 1:100 for 1h. After 5 times wash with PBS-T, Alexa Fluor 488 goat anti-mouse IgG (Thermo Fisher Scientific) were used as secondary antibodies at a dilution of 1:1000. Following five washes, nuclei were counterstained with 1 μg/ml DAPI (BD Biosciences) for 5 min in the dark. Photography was performed on an inverted fluorescence microscope (Thermo Fisher Scientific).

### Enzyme-Linked Immunosorbent Spot Assay

ELISPOT assay was performed to determine the number of M2e-specific IFN-γ and IL-4 secreting cells in spleen according to the manufacturer’s protocol (DAKAWE, China). Briefly, 7 days post the last immunization, mice were sacrificed and the spleens were harvested to prepare single-cell suspensions. Around 3×10^5^ splenocytes were seeded to each well of plates and stimulated with M2e peptides (equal amounts of M2e peptide from human, swine, and avian influenza viruses) at a final concentration of 10 μg/ml. After 32-34 hours of culture at 37°C, 5% CO_2_, the splenocytes were removed by cell-cracking buffer of ice-cold deionized water. The plates were then incubated with biotinylated antibodies followed the addition of HRP-conjugated streptavidin. After washing 5 times, the reaction was developed with AEC (3-amino-9-ethylcarbazole) substrate and stopped with flowing water. Plates were dried at room temperature and spot-counted (DAKAWE, Wuhan, China).

### Histopathologic Analyses

Mice (n=3) were immunized and challenged as described above. Five days after challenge with 5 LD_50_ of influenza A/PR/8/1934 virus, mice were sacrificed, and lungs were isolated. Lung tissues were fixed in 10% formalin, dehydrated through a graded series of ethanol, embedded in paraffin wax, and cut into 4 µm-thick sections. After deparaffinization, sections were stained with hematoxylin-eosin and observed under an optical microscope (Nikon, Japan).

### Statistical Analysis

All the statistical analyses were performed using GraphPad Prism software. Comparisons among different groups were evaluated by one-way ANOVA. In all cases, p< 0.05 was considered as statistically significant difference.

## Data Availability Statement

The original contributions presented in the study are included in the article/**Supplementary Material**. Further inquiries can be directed to the corresponding authors.

## Ethics Statement

The animal study was reviewed and approved by The Research Ethics Committee (HZAUMO-2021-0023), Huazhong Agricultural University, Hubei, China.

## Author Contributions

PT and ML designed the experiments. ML, PG, CC, and HF performed the experiments. ML, PG, WZ, and CG analyzed the data. ML, VR, and PT wrote the manuscript. PT directed the project. All authors contributed to the article and approved the submitted version.

## Funding

This work was supported by grants from National Natural Science Foundation of China [Grant No. 31870915 to PT], Fundamental Research Funds for the Central Universities [Program No. 2662019PY002 to PT], and in part by National Institute of Allergy and Infectious Diseases, National Institutes of Health [AI081726 to VR].

## Conflict of Interest

The authors declare that the research was conducted in the absence of any commercial or financial relationships that could be construed as a potential conflict of interest.

## Publisher’s Note

All claims expressed in this article are solely those of the authors and do not necessarily represent those of their affiliated organizations, or those of the publisher, the editors and the reviewers. Any product that may be evaluated in this article, or claim that may be made by its manufacturer, is not guaranteed or endorsed by the publisher.
