# Supplementary material for: Bacteriophage T4 Vaccine Platform for Next-Generation Influenza Vaccine Development
Source: Front Immunol. 2021 Oct 12;12:745625. doi: 10.3389/fimmu.2021.745625 (PMC8546227; doi:10.3389/fimmu.2021.745625)
Supplement: Supplementary file 1 [file Presentation_1.pdf]

Supporting information

Bacteriophage T4 Vaccine Platform for Next-generation Influenza Vaccine Development

Mengling Li [a,b,c,f], Pengju Guo [a,b,c,f], Cen Chen [a,b,c,f], Helong Feng [c,d], Wanpo Zhang [c], Changqin Gu [c], Guoyuan Wen[d,*] , Venigalla B. Rao[e,*], and Pan Tao [a,b,c,f*]

[a] Key Laboratory of Development of Veterinary Diagnostic Products, Ministry of Agriculture, College of Veterinary Medicine, Huazhong Agricultural University, Wuhan 430070, China.

[b] The Cooperative Innovation Center for Sustainable Pig Production, Huazhong Agricultural University, Wuhan, Hubei 430070, China.

[c] Division of Pathology, College of Veterinary Medicine, Huazhong Agricultural University, Wuhan, Hubei 430070, China.

[d] Institute of Animal Husbandry and Veterinary Sciences, Hubei Academy of Agricultural Sciences, Wuhan, Hubei 430070, China.

[e] Bacteriophage Medical Research Center, Department of Biology, The Catholic University of America, Washington, DC 20064, USA

[f] Hongshan Lab, Wuhan, Hubei 430070, China.

*To whom correspondence should be addressed. E-mail: taopan@mail.hzau.edu.cn; rao@cua.edu; wgy_524@163.com

FIGURE S1

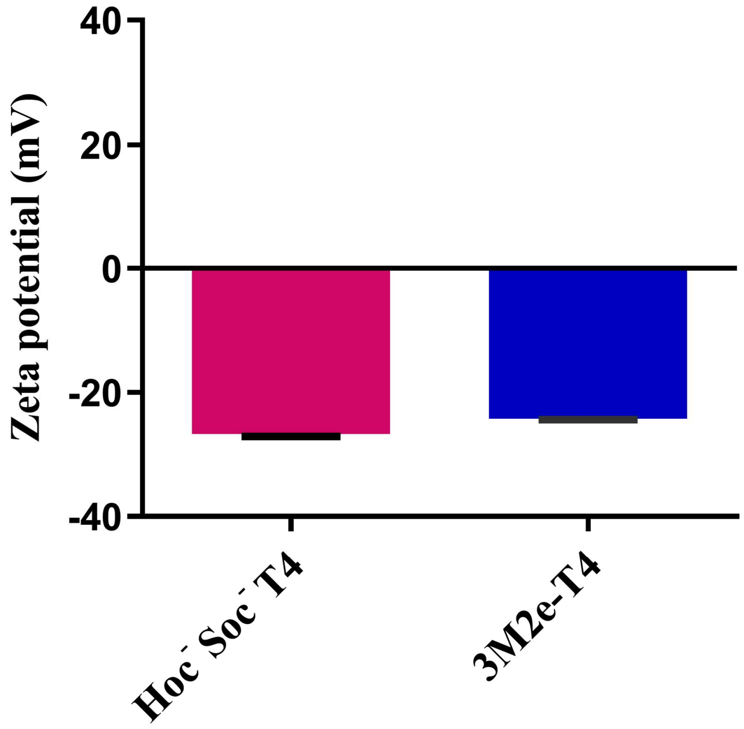

FIGURE S1. The zeta-potentials of Hoc$^-$Soc$^-$ T4 and 3M2e-T4 nanoparticles. Hoc$^-$Soc$^-$ T4 phages were purified as described in the Materials and Methods. 3M2e-T4 nanoparticles were prepared by assembly of Soc-3M2e proteins on the Hoc$^-$Soc$^-$ T4 phages. The zeta-potentials of nanoparticles were determined using Zetasizer Nano ZS (Malvern Panalytical, UK).

FIGURE S2

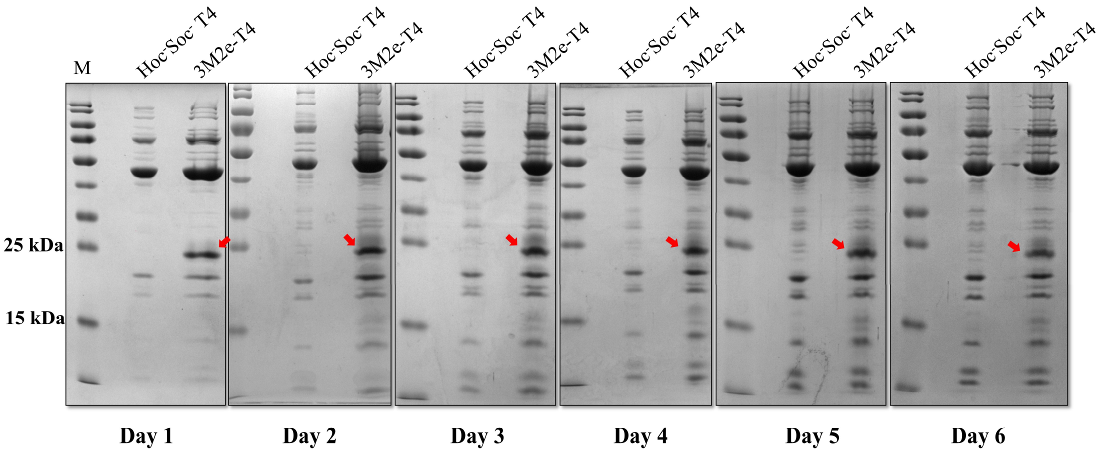

FIGURE S2. The stability of 3M2e-T4 nanoparticles stored at 4°C. The 3M2e-T4 nanoparticles were prepared as described in the Materials and Methods and stored at 4°C for 6 days. About 7×10^{10} PFU 3M2e-T4 nanoparticles were withdrawn every day and used for SDS-PAGE analysis. $Hoc^{-}Soc^{-}$ T4 phages were used as controls. Red arrows indicate the bands of Soc-3M2e protein.

FIGURE S3

A B

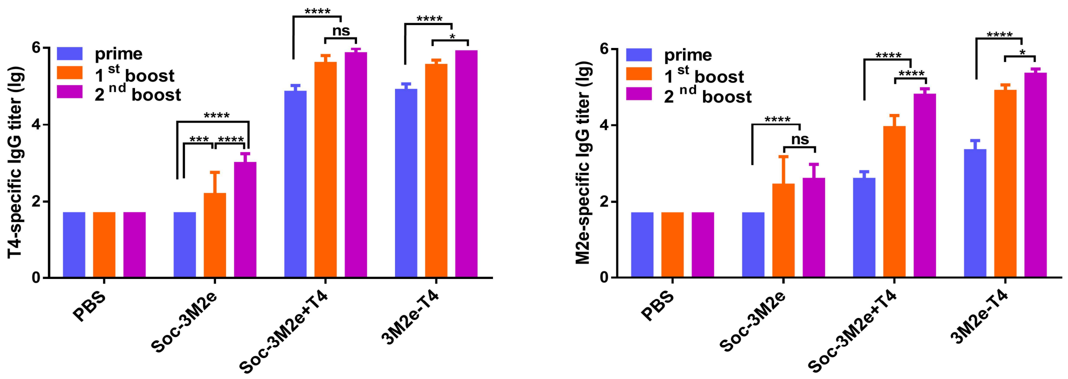

FIGURE S3. The titers of T4-specific and M2e-specific IgG. Four groups of mice were immunized with PBS, Soc-3M2e protein, the mixture of Soc-3M2e protein and Hoc$^-$Soc$^-$ T4, and 3M2e-T4 nanoparticles respectively. Sera samples were collected as indicated in Fig.2A. The titers of T4-specific **(A)** and M2e-specific IgG **(B)** were determined by ELISA. Date were shown as means±S.D. *, ***, and **** indicated $p < 0.05$, $p < 0.001$, and $p < 0.0001$ respectively (ANOVA).

FIGURE S4

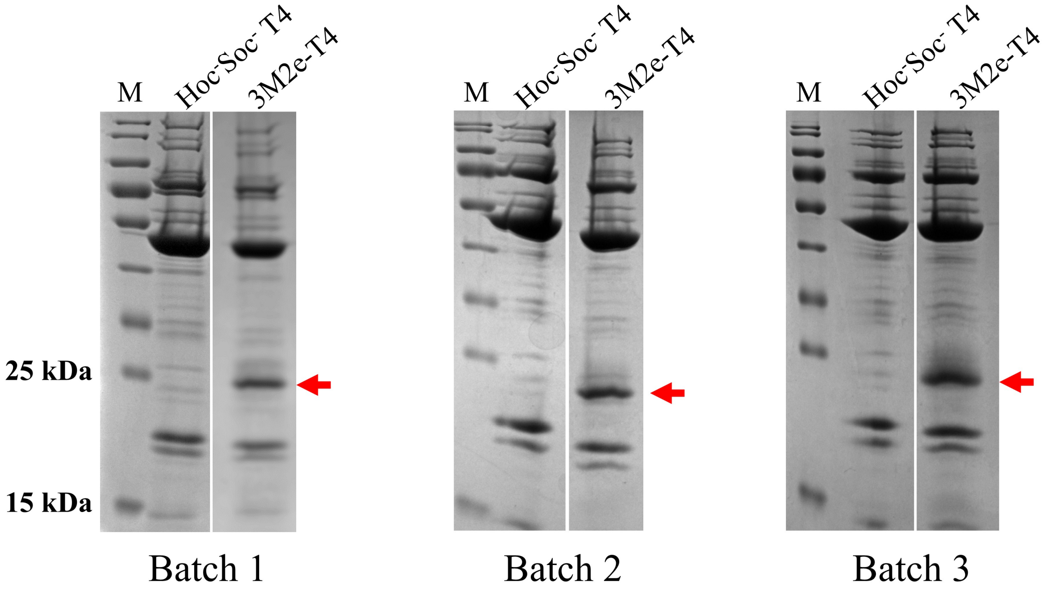

FIGURE S4. The consistency of three batches of 3M2e-T4 nanoparticles.

Three batches of 3M2e-T4 nanoparticles used for animal experiments were analyzed by SDS-PAGE for consistency before used for animal immunizations. Hoc^-Soc^- T4 phages were used as controls. Red arrows indicate the bands of Soc-3M2e protein.

Table S1. The sequence of 3M2e.

**The sequence of 3M2e**	
Nucleotide sequence (5' to 3')	GTCGAC GGC GGC AGC AGC GGC GGC AGC AGC ATG AGC CTG CTG ACC GAA GTG GAA ACC CCG ATT CGT AAC GAA TGG GGC TGC CGT TGC AAC GAT AGC AGC GAT GGC GGC AGC AGC GGC GGC AGC AGC ATG AGC CTG CTG ACC GAA GTG GAA ACC CCG ACC CGT AGC GAA TGG GAA TGC CGT TGC AGC GAT AGC AGC GAT GGC GGC AGC AGC GGC GGC AGC AGC ATG AGC CTG CTG ACC GAA GTG GAA ACC CCG ACC CGT AAC GAA TGG GAA TGC CGT TGC AGC GAT AGC AGC GAT CTCGAG
Amino acid sequence (NH2- to COOH-)	GGSSGGSSMSLLTEVETPIRNEWGCRCNDSSDGGSSGGSSMSLLTEVETPTRSEWECRCSDSSDGGSSGGSSMSLLTEVETPTRNEWECRCSDSSD

Nucleotide and amino acid sequences of M2e from human, swine, and avian influenza viruses are highlighted in brown, green, and red respectively. The flexible linkers are shown in black. SalI and XhoI cloning sites are added at 5' and 3' ends of 3M2e respectively and highlighted with underline.
